# Effects of Natural and Human-Assisted Regeneration on Landscape Dynamics in a Korean Pine Forest in Northeast China

**DOI:** 10.1371/journal.pone.0082414

**Published:** 2013-12-06

**Authors:** Fuqiang Zhao, Jian Yang, Hong S. He, Limin Dai

**Affiliations:** 1 State Key Laboratory of Forest and Soil Ecology, Institute of Applied Ecology, Chinese Academy of Sciences, Shenyang, China; 2 School of Natural Resources, University of Missouri, Columbia, Missouri, United States of America; University of Tartu, Estonia

## Abstract

Improper forest harvesting can potentially degrade forest ecosystem functions and services. Human-assisted regeneration (e.g., planting) is often used to increase the rate of forest recovery and thereby reduce regeneration failure. Seed dispersal is a fundamental ecological process that can also influence spatio-temporal patterns of forest regeneration. In this study, we investigated the relative contribution of planting and seed dispersal on forest regeneration at landscape scales. Because such influences can be further complicated by timber harvest intensity and seed availability within and around harvested area, we also evaluated the effects of those factors on forest landscape dynamics. We used the forest landscape model LANDIS to simulate the dynamics of Korean pine-broadleaf mixed forests in Northeast China. We considered three factors: timber harvest intensity (3 levels), seed dispersal and whether or not planting was used. The results showed that planting was more important in maintaining the abundance of Korean pine (*Pinus koraiensis*), a climax keystone species in this region, under the high-intensity harvesting option during early succession. In contrast, seed dispersal was more important during late succession. Korean pine can be successfully regenerated through seed dispersal under low and medium harvest intensities. Our results also indicated that effective natural regeneration will require protecting seed-production trees (seed rain). This study results provide a basis for more effectively managing Chinese temperate forests and possibly other similar ecosystems.

## Introduction

Seed dispersal is a fundamental process in natural regeneration. It can impact the rate, spatial range, and overall spatio-temporal patterns of regeneration [1-3]. It can also affect forest diversity [4], species composition [5], and plant community dynamics [6,7]. Seed dispersal can be influenced by many factors including seed size, seed source abundance, seed viability, dispersal mode, and degree of forest fragmentation [8-10]. Extensive research has been conducted on effects of fragmentation and species trait on seed dispersal [11-13]. However, little research has been done on the cascading effects of reduced efficiency of seed dispersal on natural regeneration and forest landscape dynamics. 

Besides seed dispersal, human-assistant practices such as tree planting can be used to regenerate forests [14,15]. Planting can result in rapid re-establishment of forests, reduce dependency on natural regeneration, and accelerate restoration of the ecological services provided by natural forests [16-18]. However, tree planting is often accomplished via large-scale monocultures, which are usually different from the species composition and age structure that would result from natural regeneration [18]. In addition, the costs of planting and post-planting care are high, sometimes leading to unexpected negative consequences. 

Timber harvesting, by itself, may also alter interspecific interactions. Although harvesting practices can potentially influence certain ecological processes [19], improper harvesting can also produce undesirable effects [20-22]. Due to excessive logging, the mature and old-growth forests in many regions have been converted to less desirable types, which sometimes include planted forests [23,24]. In both cases, younger and simpler forest age structures usually result [25,26]. Because young forests produce less seeds than mature forests, seed dispersal also becomes less available for natural regeneration [27,28]. Large areas of greatly reduced stand density or clear-cutting may lead to slow rate of natural regeneration [29,30] and drastically degrade ecosystem processes such as nutrient cycling, enerygy capture, biomass creation, regulation of the water cycle [31-33].

Relying on natural regeneration through seed dispersal can potentially reduce planting costs. However, effects of timber harvest intensity on seed dispersal and the actual need for planting remain largely unknown. Moreover, these questions apply to both stand and landscape scales for the following reasons: 1) both stand-level forest structure and landscape-level vegetation heterogeneity can influence seed dispersal; 2) effects of regeneration, growth, mortality, and plant competition on forest dynamics among stands, both collectively and interactively, affect landscape-level species abundance, composition, and age structure; 3) forest management plans are often designed at landscape scales that consider how human intervention influences forest landscape dynamics. For example, planting and seed harvest may directly change the natural dynamics of forest regeneration. In contrast, different cutting intensities may indirectly change this dynamic due to reduction in seed production and dispersal. These effects on landscape dynamics may amplify or mitigate each other. Exploring these questions at large spatial extents (>10^4^ ha) over long temporal periods (>100 years) thus are important to understanding successional processes and designing effective forest management plans. 

The objective of this study was to explore the relative contribution of seed dispersal and planting on landscape dynamics in a cool-temperate forest in Northeast China. We hypothesized that: 1) natural regeneration through seed dispersal may be sufficient to obtain satisfactory landscape-level forest recovery when timber harvest intensity per decade is low, i.e., is restricted to a small percentage of forest area; 2) human-assisted regeneration may be necessary when timber harvest intensity is high; and 3) the contribution of seed dispersal to forest regeneration may increase during late succession due to the cumulative effects of dispersal. We addressed the following questions: 1) Did the effects of seed dispersal and planting on forest regeneration change with timber harvest intensities? 2) Did the individual and combined effects of planting and seed dispersal change species abundance at the landscape scale? and 3) Did the relative importance of seed dispersal vs. planting on species abundance vary with harvest intensity and successional state? We tested these hypotheses by using a simulation approach that considered three factors: harvest intensity (3 levels), seed dispersal, and planting options (yes or no). 

## Materials and Methods

### Study area

**Figure 1:**
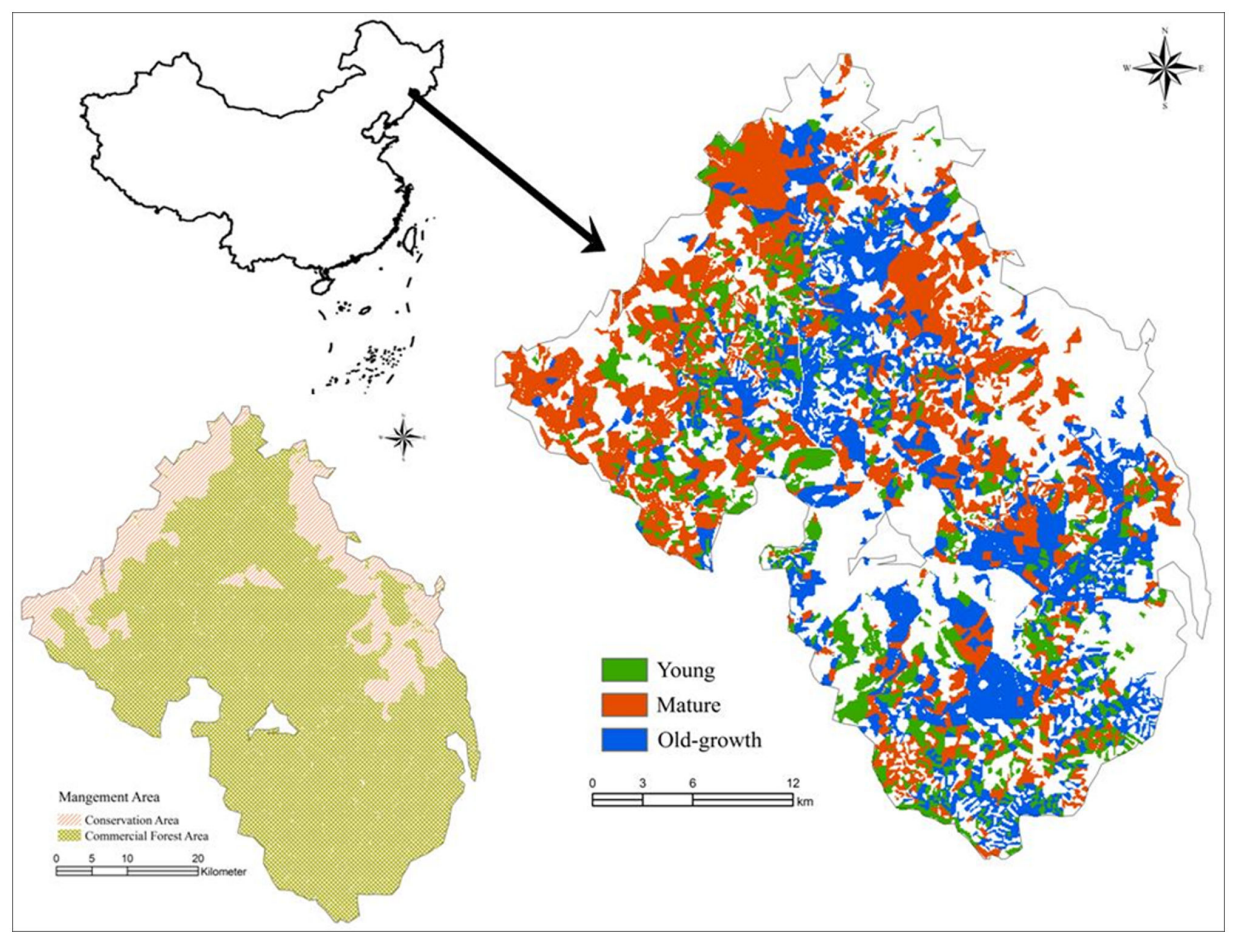
Map of the Lushui River Forestry Bureau in northeast China and the initial age-distribution of Korean pine.

The study area is located in the Lushui River Forestry Bureau (42°20′~42°40′N, 127°29′~128°02′E), a state-owned forest situated in the Changbai Mountains of Northeast China (Figure 1). The study site is located on the northwest-facing slope of the Changbai Mountains, covering about 1.3×10^5^ ha. Forests in this region are predominantly Korean pine-broadleaf mixed forests with a distinctive species composition and high biodiversity. This forest type provides important economic and ecological services to Northeast China. The forest is characterized by a continental climate, with long cold winters and short hot summers. Mean annual precipitation and temperature are approximately 894 mm and 2.9°C, respectively. Soils are classified as dark brown forest soil or Mollisols according to the American Soil Taxonomy Series. The major coniferous tree species include Korean pine (*Pinus koraiensis*) and spruce (*Picea koraiensis*). Broadleaf species include basswood (*Tilia amurensis*), maple (*Acer mono*), Manchurian ash (*Fraxinus mandshurica*), Manchurian walnut (*Juglans mandshurica*), Mongolian oak (*Quercus mongolica*), white birch (*Betula platyphylla*), elm (*Ulmus pumila*), and aspen (*Populus davidiana*). Korean pine was the most important timber species during the period of intensive clear-cutting (1970s and 1980s) and is the climax species in this region. 

Since 2000, the Chinese government has pursued a new forest management policy, called Natural Forest Conservation Programme (NFCP). Under this policy, different levels of timber harvesting intensity have replaced the previous monoculture-oriented high-intensity harvesting. The new system divides the forests of the Lushui River Forestry Bureau into two kinds of management areas: commercially harvested areas (80%) and conservation areas (20%). In conservation areas, the management goal is to maintain native biodiversity and ecosystem processes and functions. Although timber harvesting in conservation areas is prohibited, seed harvesting is allowed. The regulated reduction of timber harvesting has led to decreased economic profit. So to maintain the income of local residents, high intensity seed harvesting has been permitted by the local government, but with little consideration given to potential negative effects on seed dispersal and thus natural regeneration. Korean pine seed has been harvested with high intensity as its high economic value. The amount of pine seeds stored in the forest floor has decreased to less than 1% of that in 1970s. In turn, this has become a major factor limiting the natural regeneration of Korean pine [34].

### Experimental design

To investigate the relative effects of seed dispersal and planting on Korean pine landscape dynamics, we designed a factorial experiment that included the three harvesting intensities, two seed dispersal and two tree planting modes (Yes and No). Timber harvest intensities were set at three levels: low, medium, and high. For low intensity harvesting, the commercial harvested area is set at 3% of commercial forest area per decade. This maintains a 300-year harvest rotation, which is considered one successional cycle for the Korean pine-broadleaf mixed forest type in the Changbai Mountains. Medium level harvesting is set at 5% of commercial forest area per decade. This equates to a 200-year rotation, which provides wood production while maintaining 2/3 of the natural succession period. The highest harvest intensity is set at 10% of commercial forest area per decade. This provides maximum wood production while maintaining basic ecosystem functions and services. 

We used LANDIS, a spatially explicit forest landscape model (see next subsection), as a simulation tool. Based on the experimental design, 12 scenarios were created using LANDIS to simulate forest succession, seed dispersal, and timber harvesting. The simulation period began in 2003 and ran for 300 years. Each scenario was replicated ten times to account for stochasticity in the LANDIS model.

While recognizing that ecological effects of seed dispersal, planting, and harvesting are complex and interrelated, we limited our analysis to comparisons of Korean pine abundance and age structure. Response variables for species composition were expressed as the proportion of pixels where the species is present relative to those occurring across entire landscape.

### Description of LANDIS model

LANDIS 6.0 (http://landis.missouri.edu/), is a spatially explicit landscape model designed to simulate forest landscape change over long periods (>100 years) and across large spatial scales (>10^4^ ha). LANDIS can simulate succession, seed dispersal, species establishment, timber harvest, fire and wind disturbance, in addition to interactions among those variables. LANDIS simulates spatial processes and interactions based on raster that grid the study area into cells. Each cell is a spatial object track that includes: 1) the presence or absence of age cohorts of individual species in a binary format, 2) mean fire/wind return interval, 3) time since last fire/wind/harvest disturbance, and 4) species establishment ability for defined environments. At the site scale, species birth, growth, death, regeneration, random mortality, and vegetative reproduction are simulated at 10 year time steps for each cell. In LANDIS, it is assumed that detailed, individual tree information and within-stand processes can be simplified, allowing large-scale questions such as spatial pattern, species distribution, and disturbances to be adequately addressed. Detailed information on model design and testing is available from other sources [35,36]. In this paper only the modules that are related to our research (i.e., succession and harvest) are presented and discussed.

LANDIS simulates succession as non-spatial site-level ecological processes including establishment, growth and longevity-induced mortality. Spatial processes such as seed dispersal and disturbance-induced mortality are simulated separately. Succession at each site is a competitive process driven by species life history attributes. LANDIS also tracks the presence and absence of species’ age cohorts. Therefore, succession dynamics are simplified and simulated as birth, growth, and death processes acting on species’ age cohorts. The birth process is determined by seed dispersal and species establishment. In LANDIS, seed dispersal simulates seed travel based on a species’ effective and maximum seed dispersal distance. When a seed successfully arrives at a given site, a seedling establishment algorithm decides whether the seed can establish based on species that occur on the site and the shade tolerance ranking of the species as a seedling relative to other species occupying the site. The effective seed dispersal distance is the distance at which a seed has the highest probability (*P*>0.95) of reaching a site. The maximum seed dispersal distance is that distance beyond which a seed has near zero probability (*P*<0.001) of reaching the destination site. Once LANDIS determines that the seeds of one or more species can reach the destination site, it then selects which species are most likely to establish and grow there based on species establishment coefficients (SECs). SECs range from 0 to 1.0 and express a given species’ relative ability to grow on different site categories or land types [37]. Species with high SEC values thus have high probabilities of establishment.

In the LANDIS harvest module, the landscape is divided into several management areas (MAs). MAs are then further divided into stands, which are the basic timber harvest unit. As LANDIS tracks the presence and absence of species age cohorts, once a stand is selected for harvesting the module removes specific selected cohorts of selected species. Multiple harvest prescriptions which consider harvest area, targeted species, and age cohorts to be harvested, can be used in the same MA. However, only one harvest prescription can be applied on a stand. LANDIS can also simulate tree planting. Once planting is turned on, the model randomly selects those harvested pixels for planting based on the planting prescription (i.e., which species is designated for planting). Planting simply establishes the age of youngest cohort of a given species following the harvest of each cell. However, it does not consider the survival rate of planted seedlings.

### Parameterization of LANDIS

LANDIS simulations require several raster maps and input parameter files for model initialization. The raster maps include a forest composition map, a land type map, and a management area map. The size of pixel in the model was set at 90 m x 90 m (0.81 ha) and a 10-year time step was used to simulate all processes. The input parameter files include a species vital attribute file, a land type attribute file, and a harvest prescription file. The LANDIS model has been widely used and has been parameterized for our study area [37,38]. 

Ten major tree species were simulated in this study. They represent the most common tree species found in this area. Species vital attributes (Table 1) were estimated based on existing studies in the region [39-41] in consultation with local experts. The forest composition map was derived from the forest stand map and inventory database provided by the Lushui River Forestry Bureau. The forest stand map was converted into grid format using a geographic information system (GIS). We developed species composition and age distribution for each vegetation type based on the forest stand inventory database. We then randomly assigned each cell with a list of age cohorts based on these distributions [42]. 

**Table 1:** Vital attributes of the major tree species on the forest landscape administered by the Lushui River Forestry Bureau, China.

Species	LONG	MTR	ST	FT	ED	MD	VP	MVP	HA
*Pinus koraiensis*	300	40	4	4	50	100	0	0	120
*Picea koraiensis*	300	30	4	4	50	150	0	0	120
*Tilia amurensis*	300	30	4	2	100	100	0.1	60	80
*Acer mono*	200	30	4	3	100	200	0.3	60	80
*Populus davidiana*	150	30	2	1	-1	-1	1	0	40
*Betula platyphylla*	150	20	1	1	20	4000	0.8	50	60
*Ulmus pumila*	250	30	3	3	300	1000	0.7	60	60
*Juglans mandshurica*	250	30	2	4	50	100	0.9	60	80
*Fraxinus mandshurica*	300	30	4	2	50	150	0.1	80	80
*Quercus mongolica*	300	30	4	3	40	100	0.5	60	80

LONG: life span (years); ED: effective seeding distance (m)

MTR: age of maturity (years); MD: maximum seeding distance (m)

ST: shade tolerance (categorical classes 1 to 5, as class 5 indicates most tolerant)

FT: fire tolerance (categorical classes 1 to 5, as class 5 indicates most tolerant)

VP: vegetative reproduction probability (0–1)MVP: minimum age of vegetative reproduction (years)HA: harvest age

We defined seven land types mainly based on terrain conditions. The first land type represented non-forested area. The remaining six forested land types were: valley bottom, gentle slope, terrace, shady slope, sunny slope, and steep slope. SECs were empirically derived from species eco-physiological attributes [40,41,43] and previous LANDIS research studies in this region [38,44]. Forested land was classified as either Conservation Area or Commercial Forest Area based on the NFCP. Selection harvesting and planting were simulated in the Commercial Forest areas. Areas selected for harvesting were set to different proportion of the MA harvested per decade in Commercial Forests as defined above i.e., 3%, 5%, and 10% to represent low, medium and high harvest intensities, respectively. All species were candidates for harvesting in the simulations. However, only age cohorts that attained old-growth status (e.g. Korean pine>120 years) were harvested. After simulated harvesting, all species were randomly selected for planting together in the cells to be planted. Planting intensities were the same as those defined for harvesting intensities. None of the Conservation Areas were harvested or planted in the simulation.

### Data analysis

The simulation results were analyzed using a two-factor univariate analysis in the General Linear Model (SPSS 16.0) program, which was applied to each harvest intensity level within each simulation periods. The dependent variables (percent coverage of Korean pine under each scenario with ten replications) were tested for normality and homogeneity of variances in the residuals. Independent variables (planting and seed dispersal) were both fixed factors with two levels.. Type III sums of squares derived from the univariate analysis was used to quantify the relative importance of planting, seed dispersal and their interaction with Korean pine abundance at the landscape scale. Higher type III sums of square values indicated stronger contributions to changes in Korean pine coverage at the landscape scale. The actual type III sums of square values for planting, dispersal and their interactions were comparable within one statistical model, but not necessarily between two or more models. We therefore used proportions of actual type III values for comparing differences among the relative importance for different simulation periods. Because forest management planning is often used for guiding future management within 100 years, we only compared the relative contributions of planting, seed dispersal, and their interactions among year 0-30, 40-70 and 80-100 simulation periods.

## Results

### Effects of seed dispersal and planting on Korean pine regeneration

**Figure 2:**
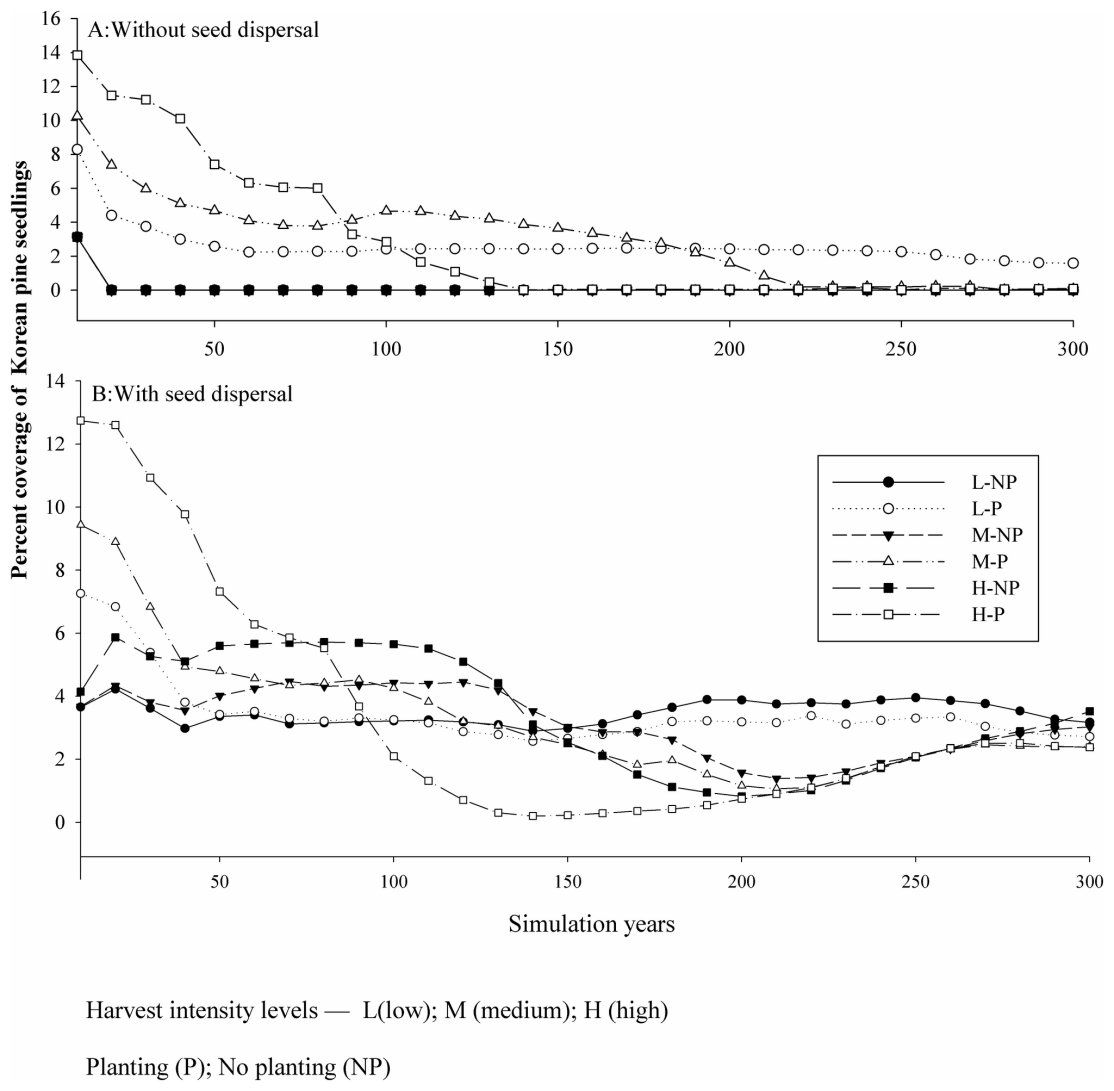
Abundance (i.e., percent coverage) of Korean pine seedlings in relation to independent variables.

Since our research focus on the effects of seed dispersal and planting on regeneration, we only analyzed the relative contribution of those factors on the abundance (i.e., percent coverage) of Korean pine seedlings. The results showed that the effect of planting decreased as simulation periods increased (Figure 2). After 200 simulation years, the abundance of Korean pine seedlings (less than 10 years old) resulting from seed dispersal was similar under planting and no-planting scenarios. However, under the high harvest intensity option, planting in the absence of seed dispersal could not maintain an abundance of pine seedlings. In no-planting scenarios, an abundance of pine seedlings was sustainable throughout the simulation periods provided there was also seed dispersal (Figure 2b). The results showed that only planting increased the abundance of Korean pine seedlings during the 50-year simulations. But even after 150 simulation years, the effect of planting on pine seedling abundance was minor when seed dispersal was considered (Figure 2b). After 100 simulation years, the effects of dispersal on pine regeneration increased. The effects of planting and seed dispersal on pine regeneration were related with harvest intensity. Under higher harvest intensities, pine seedling abundance increased under all simulation scenarios during early simulation periods. Under high harvest intensities the abundance of Korean pine seedlings sharply decreased after 100 simulation years even when planting was turned on (Figure 2). After 100 simulation years, seedling abundance was highest under the no-planting/low harvesting option when combined with seed dispersal.

### Effects of seed dispersal and planting on Korean pine abundance

**Figure 3:**
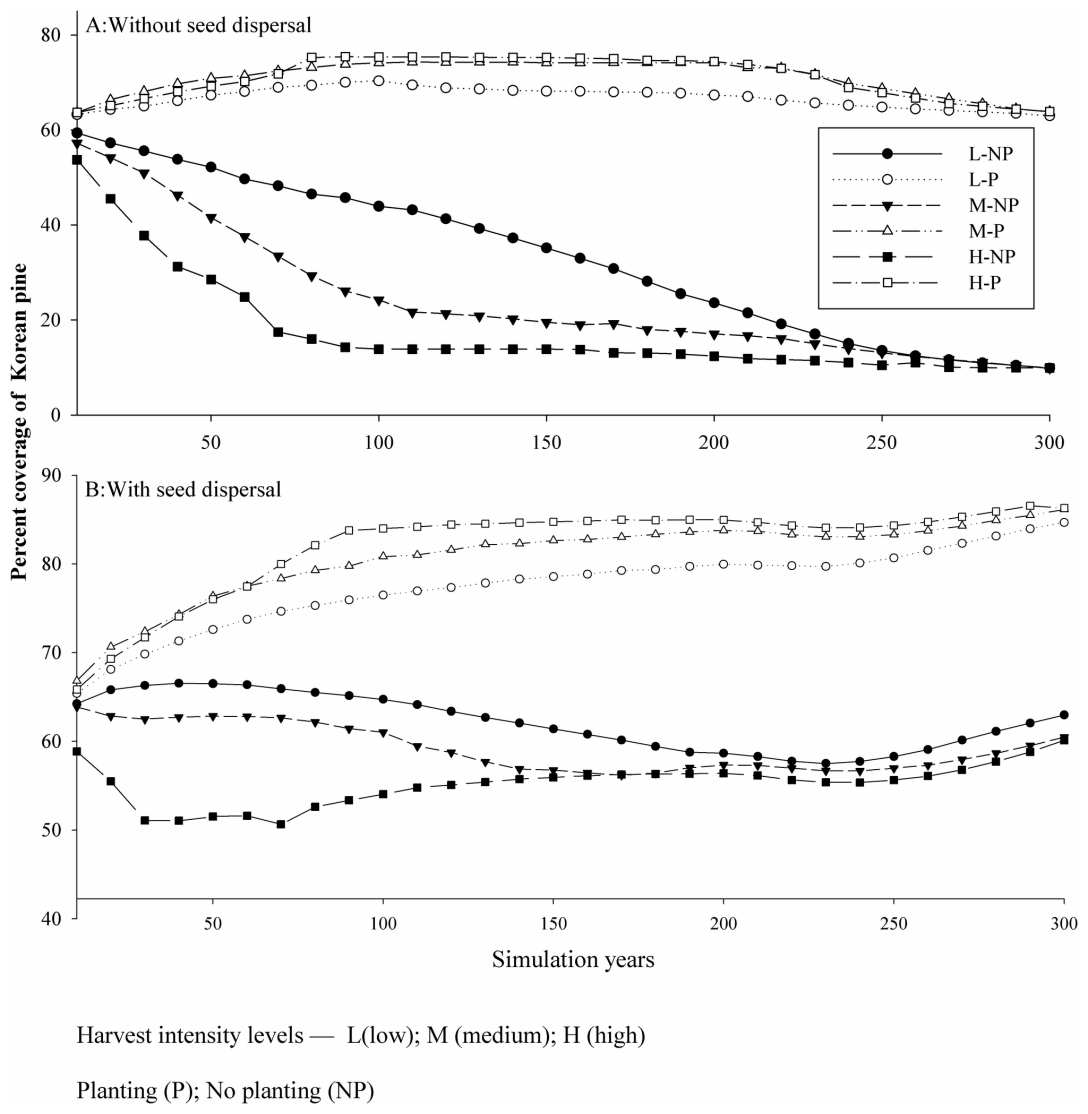
Abundance (i.e., percent coverage) of Korean pine in relation to independent variables.

The percent coverage of Korean pine was always higher with than without planting regardless of seed dispersal (Figure 3). In contrast, harvest intensity did not greatly influence the percent coverage of Korean pine when planting was used. For the ‘no planting’ scenarios, the percent coverage of pine was lowest when there was no pine seed dispersal, which in that case coverage declined from 59% to 10% from the beginning to the end of the 300-year simulation period (Figure 3a). Percent coverage was higher with seed dispersal independent of planting, which ranged from 60% to 64% (Figure 3b). Without planting, percent coverage of pine decreased with increasing harvest intensity. For low and medium harvest intensities, percent coverage showed little change. Under the high harvest intensity, the coverage was greater than 50% and remained relatively stable during simulations, indicating that under that condition seed dispersal was also important for maintain species abundance (Figure 3b). 

**Figure 4:**
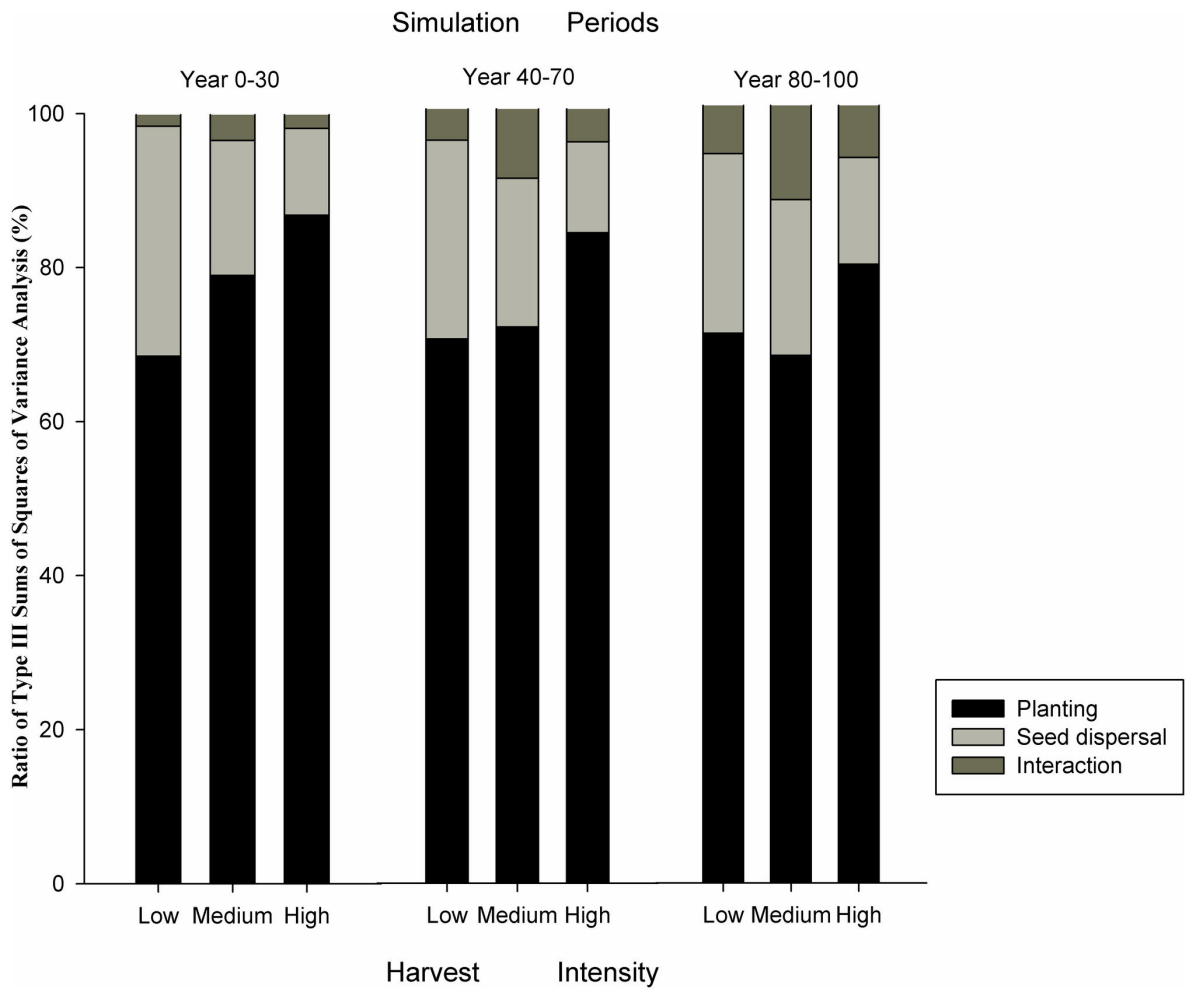
Abundance (i.e., percent coverage) of Korean pine based on type III sums of square values (fixed model) that were explained by independent variables.

The type III sums of square values in the statistical models directly reflect the relative importance of planting, seed dispersal, and their interaction on predicted Korean pine abundance at the landscape scale over long time periods. Results showed that planting was more important than seed dispersal in maintaining the area of pine under all harvest intensities. Moreover, the importance of planting increased with increasing harvest intensity (Figure 4). For the low harvest intensity, planting contributed to about 60% of the variation in the simulated Korean pine coverage, whereas seed dispersal accounted for about 35%. For the medium harvest intensity, planting contributed more than 65% to the percent of the area in pine, whereas the contribution of seed dispersal decreased to less than 25%. The pattern under high intensity harvesting mirrored that of the medium harvest intensity and the contribution of planting in that treatment was highest among the three levels of harvesting. The results also showed that the contribution of planting decreased with increasing length of simulation, i.e. planting was more important during early periods (0-30 years) than in later periods (80-100 years). In contrast, the contribution of the interaction of planting and seed dispersal increased with simulation time (Figure 4).

### Spatial pattern of age structure

**Figure 5:**
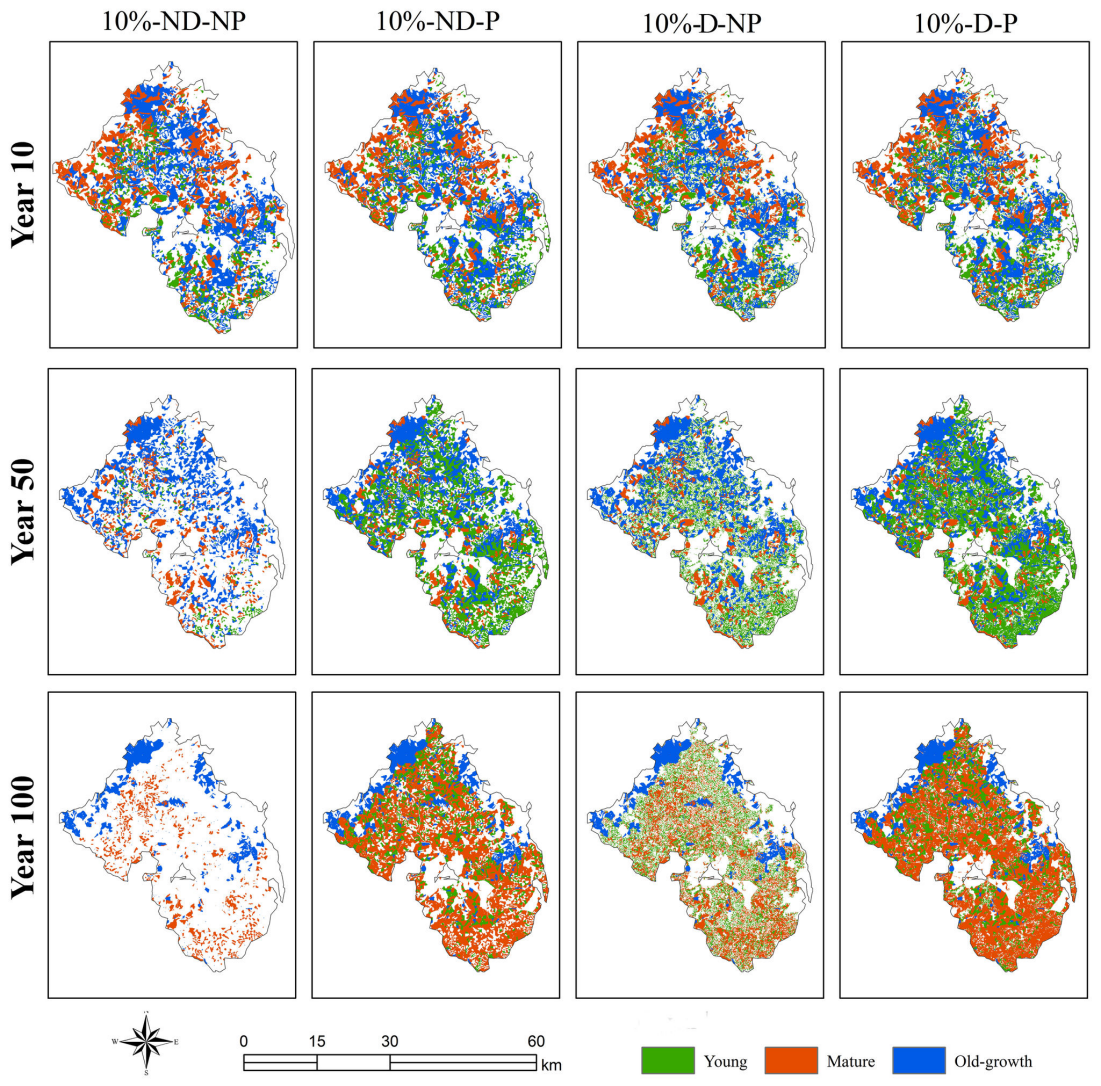
Spatial pattern of Korean Pine age structure under the high harvest intensity at simulation year 10, 50 and 100 under four scenarios extracted from one representative simulation: a) ND-NP (no seed dispersal and no planting), b) ND-P (no seed dispersal with planting), c) D-NP (with seed dispersal but no planting), and d) D-P (with seed dispersal and planting).

The Lushui River Forest Bureau classifies Korean pine into young (< 60 years), mature (60-120 years), and Old-growth (>120 years) age groups. By analyzing the spatial pattern of age structure under high harvest intensity scenarios, we found that the spatial pattern of different age groups under planting were aggregated and with reduced amount of edge (Figure 5). The abundance of young age group was highest at medium simulation period, while the mature group was highest during the late simulation period. Under the ‘no planting with seed dispersal’ scenario, spatial pattern of age structure appeared to be dispersed. The distribution of different age groups became increasingly homogeneous as edge increased. The distribution of residual old-growth Korean pines was almost exclusively limited to Conservation Area, indicating an insufficiency of pine for meeting the age requirement for harvesting during the late simulation period.

### Effects of Korean pine seed dispersal on abundance of broadleaf species in Conservation Areas.

**Figure 6:**
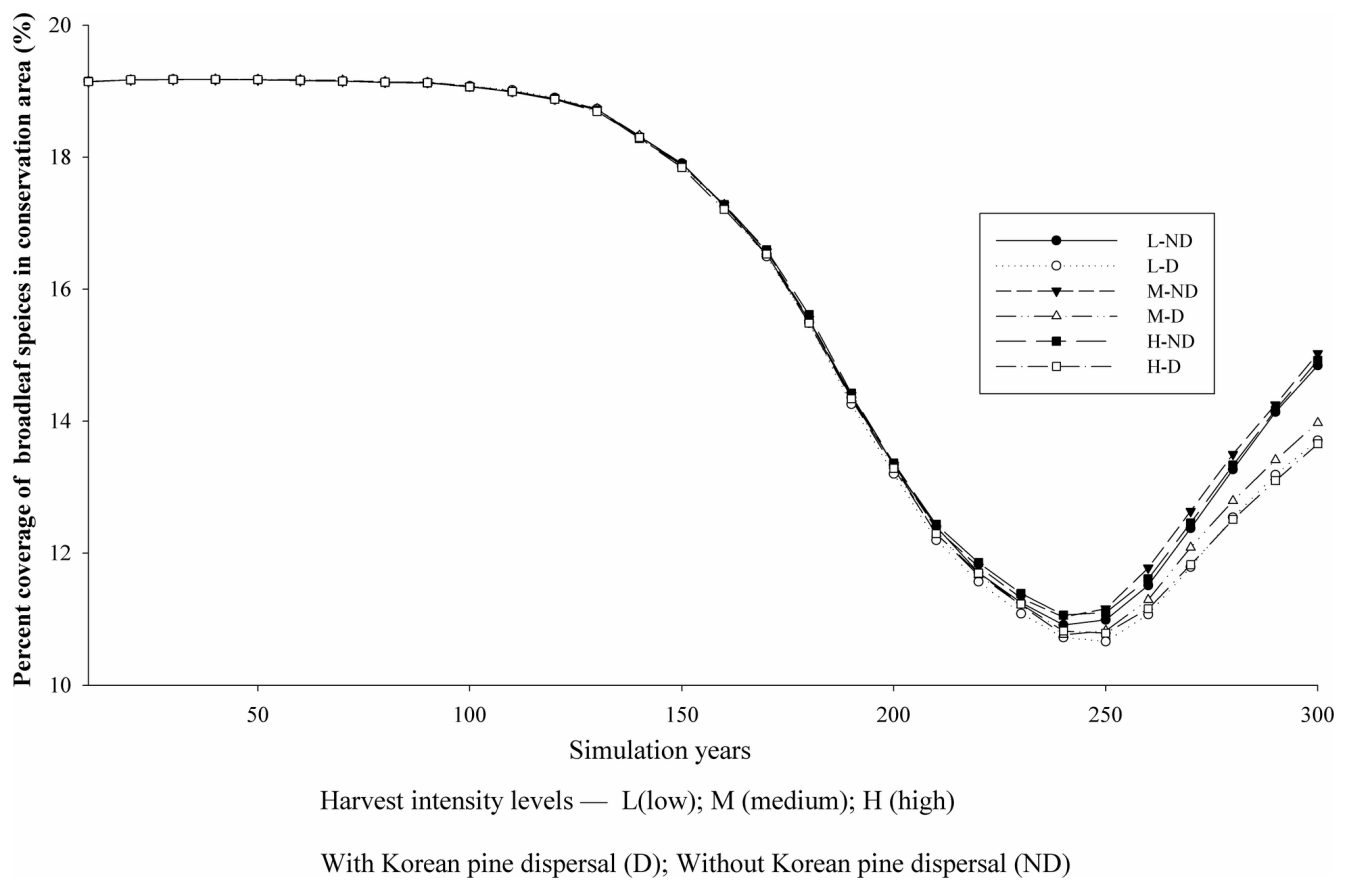
Abundance (i.e., percent coverage) of broadleaf species in relation to independent variables.

To eliminate confounding effects of Korean pine harvest and planting on other species abundance, we examined broadleaf tree species abundance only in the conservation areas where timber harvest and planting was strictly prohibited. The results showed that the dynamics of broadleaf species abundance before 200 simulation years was almost same among each scenario. It was decreased from 100 to 200 simulation years, and then increased from 200 to 300 simulation years. The abundance of broadleaf species was higher under scenarios without Korean pine seed dispersal than those with Korean pine seed dispersal after 200 simulation years. Harvest intensity in the commercial areas can also influence the abundance of broadleaf tree species in the conservation areas. The abundance was highest under medium harvest intensity while lowest under low harvest intensity (Figure 6). 

## Discussion

### Management implication

Korean pine reaches maturity at a later age relative to other species in the region and may live up to 300 years. To escape from the ripe cone, pine seeds usually require help from animals. Thus Korean pine relies mainly on animals for natural regeneration [45-47]. In turn, these animals also consume the seeds of Korean pine [48]. Stand structure favorable to natural regeneration by seed dispersal also provides habitats for animals. However, before year 2000, forests in the Changbai Mountains were primarily harvested at high intensities. Thus, most of the original broad-leaved Korean pine forest has been converted to other types of secondary forests and plantations [49]. This has also led to the fragmentation of forest landscapes [50]; and the age structure of the remaining forests has become younger and simpler [51], which in turn has further reduced seed production. Because the dispersal agents mainly feed on Korean pine seeds, reduced seed rain also reduces the abundance of those animals, which further limits Korean pine natural regeneration. Due to the high economic value of Korean pine seed, the harvesting of seeds has increased in the last ten years, leading to a severe depletion of seed stock. Landscape-scale recovery is often limited by seed rain, which is particularly true for Korean pine in the Changbai Mountains. Furthermore, most of Korean pine cones grow in the upper canopy where collection by humans often leads to branch breakage. The results wound may further reduce seed production, presumably with negative consequences for regeneration and natural landscape dynamics [52].

The results of our study indicate that human-assisted regeneration is needed when harvest intensity is high and a goal is to increase the area of young Korean pine. However, this may not necessarily lead to increased numbers of seeds available for regeneration, since the harvesting of mature trees contributes to a decrease in seed rain. We found that the effects of self-renewal became more important at the late succession stage after harvesting. This was because Korean pine has only a relatively short seed dispersal distance, the effect of seed dispersal related to increasing Korean pine seedling abundance was not obvious during the early simulation period. Our results are consistent with the research of others in this region. He et al. [44] found that Korean pine harvesting may produce long-term alterations at the landscape scale that persist from decades to centuries. International studies have suggested that the lack of seed resources combined with landscape fragmentation work together to influence seed dispersal and are thus major factors hindering forest restoration [9,10]. Our results suggest that forest management in the future should consider reducing the intensity of Korean pine harvesting as well as protecting the existing mature and old-growth forests as both sources of seeds and as habitat for animal dispersers. Under the ‘with seed dispersal’ scenario, Korean pine can be successfully regenerated without planting by using low and medium harvest intensities. This result thus provides a baseline value for regenerating and restoring of Chinese temperate forest and similar ecosystems. 

Our results also demonstrated that planting will be necessary for managing forests over short time periods and/or under high harvest intensity. Planting effectively increased the abundance of Korean pine in the study area, thereby further reducing the pressure to harvest virgin forest as well as increasing the amount of seeds available for natural regeneration. However, the cost of planting is high. Reducing seed harvest intensity can mitigate planting cost. If high intensity seed harvest continues, seed production of Korean pine will be reduced to a negligible level thereby reducing both forest health and economic benefits. Although humans can, for a while, reap economic benefits from forests managed in that way, we are simultaneously mortgaging forest health and ecological services. From analyzing of the abundance of broadleaf species in conservation areas, we found that increased Korean pine seed dispersal decreased the regeneration of other species, which in turn can change landscape-level forest composition and structure. The effects of Korean pine seed dispersal on broadleaf species abundance began to emerge after 200 simulation years, suggesting such effects were slow and cumulative. Harvest in the adjacent commercial areas also may lead to shifts in the species composition and abundance of mother trees for dispersing seeds into conservation areas. This indicates that forest management should consider not only the species and management area separately but also coexisting species and neighboring areas. 

### Model limitations

Our simulation results may be affected by the limitations of LANDIS. For example, the species establishment coefficient (SECs) we used were set as constant value throughout the simulations. However, harvesting may lead to increased light, which in turn may enhance the survival probability of broadleaf trees, which compete with Korean pine. Hence, the SEC of Korean pine may change with the alteration of environmental conditions resulting from timber harvesting-factors which were not incorporated into the LANDIS model. LANDIS also does not consider the survival rate of planted seedlings, which could lead to further inaccuracies of simulated tree density. However, LANDIS only tracks the presence or absence of species within a pixel and our analysis only focused on the coverage of Korean pine. Since, the survival rate of pine cannot be zero, simulated planting will most likely indicate the presence of the planted species. Hence, this limitation should not greatly affect our interpretation of simulation results. Although the methods used in the study are somewhat theoretical, the results provide evidence of the relative effects of planting and seed harvesting under the management practices considered. Study results thus offer useful insight on how to manage these forests at the landscape level with respect to planting and seed dispersal. The results especially emphasize the strong negative relationship between seed harvesting of Korean pine and its regeneration, and thus the practices need to mitigate this effect.
